# Thoracic Radicular Pain Caused by Extravertebral Gas and Fluid Collections Associated with Osteoporotic Vertebral Fracture Containing a Vacuum Cleft

**DOI:** 10.1155/2019/4284217

**Published:** 2019-02-28

**Authors:** Kazunobu Kida, Toshikazu Tani, Tateo Kawazoe, Michio Toda

**Affiliations:** ^1^Department of Orthopaedic Surgery, Kubokawa Hospital, 902-1 Mitsuke, Shimanto-cho, Takaoka-gun, Kochi 786-0002, Japan; ^2^Department of Orthopaedic Surgery, Kurashiki Medical Center, 250 Bakuro-cho, Kurashiki City, Okayama 710-8522, Japan

## Abstract

The present study documents a phenomenon, which has received little attention despite its potential clinical importance. An 87-year-old woman presented with barely reported extravertebral gas and fluid collections probably originating from the contents of the adjacent cleft within the T10 collapsed osteoporotic vertebra. Her chief complaint was intractable pain radiating over the left thorax suggestive of intercostal neuralgia. The pain intensified when sitting up from a lateral decubitus position, correlating with a posture-related radiologic change of the intravertebral cleft, which appeared with a decubitus position and disappeared with a sitting position. Because these extravertebral collections were located where the 10th thoracic nerve root just exits the intervertebral foramina, her chest pain of a posture-dependent nature most likely resulted from nerve root compression by extravertebral gas and fluid forced out of the vertebral cleft. Posterior spinal fusion with pedicle screw instrumentation resulted in a complete resolution of the chest pain with disappearance of the extravertebral gas and fluid accumulations. An awareness of the possibility that the intravertebral cleft could communicate with the extravertebral space close to the nerve root will help avoid neurologic complications caused by bone cement leakage during vertebroplasty.

## 1. Introduction

A radiolucent area within the collapsed vertebra seen after osteoporotic vertebral fracture (OVF) usually contains gas and/or fluid, not necessarily representing a true vacuum, despite the common designation, intravertebral “vacuum” phenomenon [1] or the intravertebral “vacuum” cleft (IVC) [2].

As previously demonstrated, in some cases, in which the intravertebral and intradiscal clefts coexisted, the gas and/or fluid could migrate between the two adjacent clefts through the fractured endplate [1, 3]. Some reports also showed that symptomatic gaseous cysts could arise in the epidural or intradural space, presumably originating from the adjacent degenerated intervertebral disc [4, 5]. However, to the best of our knowledge, only a single study in the past reported on the radiologic appearance of extravertebral gas bubbles and edematous effusions secondary to the OVF containing IVC [6]. Unlike a simple radiologic observation in the previous report, the current study documents a patient who developed an intractable chest pain, not responding to conservative therapy, as a sign of the T10 nerve root compression by the localized extravertebral gas and fluid collections associated with the IVC inside the T10 collapsed vertebral body. The patient and her family provided written informed consent for publication of this case report and accompanying images.

## 2. Case Report

An 87-year-old woman with an intractable pain radiating over the left chest, suggestive of intercostal neuralgia, was referred to our spine center for further evaluation and management. Five weeks earlier, she experienced a ground-level fall, causing severe back pain. According to the initial evaluation conducted elsewhere, she was diagnosed as having OVF of the T10 vertebral body and managed conservatively with a brace and pain medication for one month. Her chief complaint gradually changed from bilateral back pain to an aching pain extending to the left thorax before her first visit to our hospital. What is noteworthy about the history of this case is that 8 years previously, she had had a painful OVF of the T12 vertebral body at the age of 79 years, which was treated with vertebroplasty using hydroxyapatite (HA) blocks and posterior pedicle screw instrumentation followed by iliac bone graft fusion from T11 to L1 at another hospital [7].

On clinical examination at our hospital, the patient had no neurologic deficits, complaining of nothing but reporting a position-dependent severe chest pain on the left side, which intensified when sitting up from a supine position. Imaging studies revealed neither rib fractures nor abnormal findings of the chest cavity as a possible cause of the chest pain.

**Figure 1:**
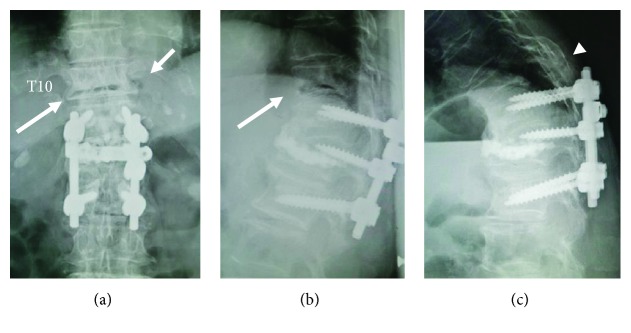
Preoperative thoracolumbar spine radiographs in the supine (a, b) and sitting (c) positions, showing intravertebral vacuum cleft (long arrows), extravertebral gas (short arrow), and the fracture line of T10 extending to posterior fusion mass bone (arrow head) grafted 8 years ago.

**Figure 2:**
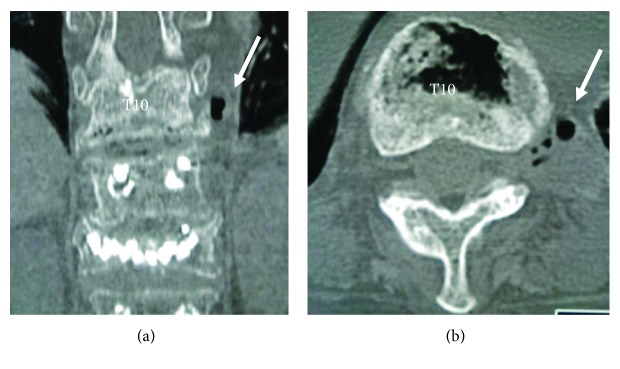
Preoperative coronal (a) and axial (b) computed tomographies of the thoracic spine obtained in the supine position, more clearly showing the gas and fluid collections (arrows) just below the left 10th rib head in (a) and the T10-T11 extraforaminal zone in (b).

On the plain radiographs of the thoracic spine (Figure 1), the anteroposterior (AP) views and the lateral views taken in the supine position showed the IVC within the T10 collapsed vertebral body as a gas-like radiolucent area, which disappeared on the lateral view obtained in the sitting position (i.e., the opening-closing phenomenon) most likely indicating an OVF of nonneoplastic and noninfectious origin [8, 9]. The lateral radiograph in the sitting position also demonstrated that the fracture line extended through the posterior fusion mass bone, grafted 8 years ago, indicating a potentially unstable flexion-distraction injury. Close observation of the AP radiograph obtained in the supine position revealed a gas-like radiolucency at the extravertebral space just lateral to the T10 collapsed vertebral wall ipsilateral to the side of her chest pain. The computed tomography (CT) scans, which have a higher sensitivity in detecting gas than radiography and magnetic resonance imaging (MRI) scans, more clearly showed an accumulation of gas located just below the head of the left 10th rib, the place radiologically termed the T10-T11 extraforaminal zone (Figure 2).

**Figure 3:**
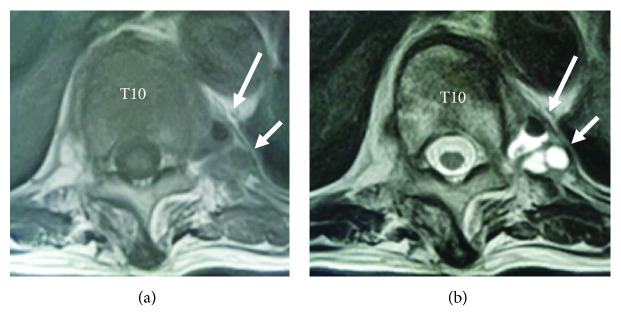
Preoperative axial T1-weighted (a) and T2-weighted (b) magnetic resonance imaging, showing the gas (long arrows pointing T1-low and T2-low signal intensity areas) and fluid (short arrows pointing T1-iso and T2-high signal intensity areas) collections just below the left 10th rib head.

In terms of the IVC contents of radiolucency on the plain radiographs, sagittal MRI revealed a gas-like signal void within the T10 collapsed vertebral body. Corresponding to the extravertebral area of radiolucency shown by the AP radiograph and the CT, both T1- and T2-weighted axial MRI showed combined gas-like and fluid-like signal intensities; i.e., a gas-like low signal intensity area with a fluid-like iso-signal intensity area on the T1-weighted image and a gas-like low signal intensity area with a fluid-like high signal intensity area on the T2-weighted image (Figure 3).

All these findings from the imaging studies suggested that the patient's chest pain probably resulted from T10 nerve root compression at the T10-T11 extraforaminal zone by a mixed accumulation of gas and fluid, which most likely originated from the adjacent IVC within the collapsed T10 vertebral body as a result of the “force pumping mechanism,” as previously postulated [6].

**Figure 4:**
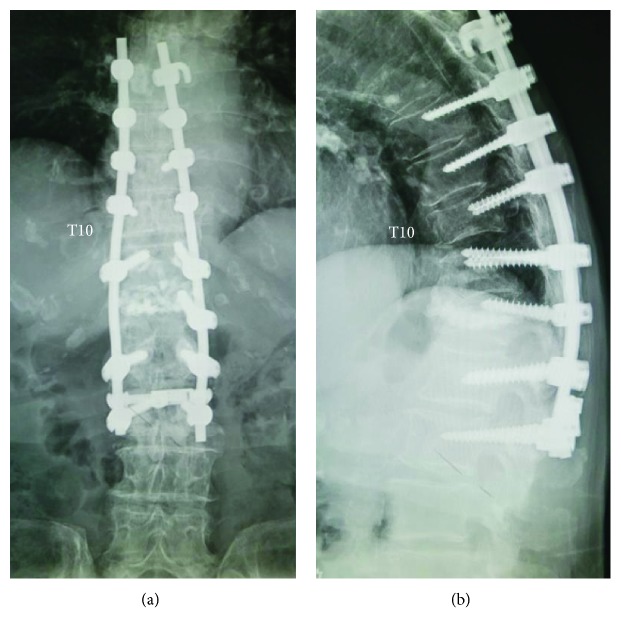
Anteroposterior (a) and lateral (b) radiographs of the thoracolumbar spine at the 2-year follow-up after surgery, showing posterior spinal fusion from T7 to L2 with pedicle screw instrumentation performed for the T10 osteoporotic vertebral fracture involving three columns.

Because of the unstable nature of the fracture involving all structural components from anterior through posterior spinal columns, in addition to her persistent disabling pain not responding to conservative therapy, we carried out posterior spinal fusion from T7 to L2 with pedicle screw instrumentation followed by iliac bone graft (Figure 4). Immediately after the surgical stabilization, the patient reported a complete resolution of the pain. This patient had no history of antiosteoporosis medications before the referral to our spine center. We administered pharmacological treatments postoperatively, consisting of teriparatide injections initially, followed by oral bisphosphonates. At a 2-year follow-up, she remained asymptomatic, and CT scans showed neither a gas accumulation nor a fluid collection both inside and outside of the T10 vertebral body.

## 3. Discussion

The IVC, initially shown by plain radiographs as a radiolucent space within the affected vertebral bodies after OVF, has been much more commonly identified by CT and MRI than previously thought. In clinical practice, the IVC, if demonstrated radiologically, serves as a fairly reliable sign that the vertebral collapse is not due to spinal neoplasm or infection [8, 9]. The presence of IVC usually indicates fracture nonunion; many studies showed a painful posture-related change of IVC, tending to widen with extension or supine position and narrow with flexion or an upright position. More recently, the advent of a new surgical technique for OVF, percutaneous vertebroplasty, has drawn increasing attention from spine surgeons to the IVC, which they need to fill with polymethylmethacrylate (PMMA) by injection with special care to avoid heat injury of the nerve by its leakage outside the vertebral body during vertebroplasty [10–12].

The current report described a patient who developed an intractable chest pain unilaterally associated with the T10 OVF containing the IVC. The pain intensified at the moment of sitting up from a decubitus position. This posture-dependent change of the pain intensity correlated with the radiographic changes of the IVC within the T10 collapsed vertebral body, appearing in a decubitus position and disappearing in a sitting position.

Importantly, we found the extravertebral small areas of gas-like radiolucency near the head of the 10th rib, ipsilateral to the side of the patient's chest pain, shown by the plain AP radiograph and more clearly by the CT. This location corresponded to the extraforaminal zone, where the 10th thoracic nerve root just exits the T10-T11 intervertebral foramina. On MRI, the signal intensities of the contents of these extravertebral radiolucent areas indicated a mixture of gases and fluids, which supports the MRI evidence commonly shown for the contents within the IVC.

To our knowledge, only one other study by Coulier [6] reported on the radiologic observation of extravertebral gas bubbles and edematous effusions associated with the OVF without any mention of the clinical symptoms. Our findings from the imaging studies coupled with the position-dependent change of the patient's pain intensity suggested that the chest pain most likely resulted from T10 nerve root compression by the extravertebral gases and fluids, which, as postulated by Coulier, could be forced out of the IVC as a consequence of increased pressure inside the IVC from the negative pressure values in the supine position to the positive values in the sitting position, the phenomenon called “force pumping mechanism.” The T10 radicular lesion may have resulted from instability of the T10 fractured vertebral body itself, but in our case, there was a lack of radiological evidence of compressive bony fragments to suggest this possibility.

To treat this elderly patient through surgery, we opted to employ a long posterior spinal fusion from T7 to L2 with pedicle screw instrumentation, not only because the patient had had a posterior spinal fusion at T11-L1 in the past but also because she had an unstable OVF involving three columns. Had it not been for the possible communication between the IVC within the T10 collapsed vertebral body and the extravertebral space close to the T10 nerve root, we could have added the T10 vertebroplasty using bone cement without concern for iatrogenic damage to the nerve root.

The fact that the extravertebral extrusion of gas and/or fluid from the IVC has rarely been reported is somewhat counterintuitive. This is because vertebral fractures, if simply accompanied by the surrounding soft tissue disruptions, as is commonly the case, involving the periosteum and the ligamentous structures attached to the fractured vertebral body surface, could communicate with the extravertebral spaces. This dissociation may suggest that the communication between inside and outside of the IVC either requires some additional key factors for its generation or escapes radiological detection by casual imaging studies without dynamic observations or both. Regardless of the underlying mechanisms of this phenomenon, its incidence rate requires further confirmation.

## 4. Conclusion

This case report indicates that the cleft within the collapsed osteoporotic vertebra could communicate with the extravertebral space close to the nerve root. An awareness of this possibility underscores the need for adequate imaging studies before vertebroplasty and kyphoplasty, which, although considered minimally invasive and low-risk procedures, may place the nerve roots at risk with bone cement leakage not only into the epidural space but also at the extraforaminal zone.
